# Patterns of domestic violence and alcohol consumption among women and the effectiveness of a brief intervention in a household setting: a protocol study

**DOI:** 10.1186/s12905-015-0236-8

**Published:** 2015-09-24

**Authors:** Carla Ferreira de Paula Gebara, Cleusa Pinheiro Ferri, Lelio Moura Lourenço, Marcel de Toledo Vieira, Fernanda Monteiro de Castro Bhona, Ana Regina Noto

**Affiliations:** Department of Psychobiology, Research Center on Health and Substance Use (NEPSIS), Universidade Federal de São Paulo (UNIFESP), Botucatu St., 862-First floor, São Paulo, SP 04023-062 Brazil; Department of Psychology, Center for Studies on Violence and Social Anxiety (NEVAS), Universidade Federal de Juiz de Fora (UFJF), Juiz de Fora, MG Brazil; Department of Statistics, Universidade Federal de Juiz de Fora (UFJF), Juiz de Fora, MG Brazil

**Keywords:** Domestic violence, Alcohol, Brief intervention, Women

## Abstract

**Background:**

Domestic violence and harmful alcohol consumption are considered major public health problems worldwide. These phenomena often co-occur, and they share several risk factors. Nevertheless, few in-depth studies have supported integrated interventions for both phenomena, in particular among Latin American women. This project will study the consumption of alcoholic beverages among women and its relationship with patterns of domestic violence; furthermore, it will assess the effect of a brief intervention (BI) aimed at modifying these behaviors using a community household sample.

**Methods/design:**

This project is divided into two studies. Study 1 will employ a cross-sectional observational design and will be conducted using a household sample of adult women (approximate sample size = 1600) to assess harmful alcohol consumption and domestic violence patterns. Study 2, will be a randomized clinical trial based on specific cases from Study 1, assessing the effect of a brief intervention on women who exhibit harmful levels of alcohol consumption (AUDIT ≥ 8). Approximately 73 women will be assigned to one of two groups, either a treated group (TG) or a control group (CG). A sociodemographic questionnaire, a questionnaire concerning general health and substance use, and four other standardized instruments (i.e., the Alcohol Use Disorder Identification Test [AUDIT; used to investigate problems related to alcohol consumption], the Center for Epidemiologic Studies Depression Scale [CES-D; used to measure depressive symptoms], and the Revised Conflict Tactics Scales and Parent–child Conflict Tactics Scales [CTS2 and CTSPC; used to obtain information on violence among couples and between parents and children, respectively]) will be used to collect data.

**Discussion:**

The study protocol will employ a household survey of a representative sample from a neighborhood in a middle income country, where well-conducted household surveys remain rare. The present work represents a step toward a better understanding of violence in women’s lives and its interaction with alcohol consumption and expands the discussion on the potential strategies for public health actions seeking to prevent both domestic violence and harmful alcohol consumption.

**Trial registration:**

Brazilian Clinical Trials Registry: RBR-7rjt4t. Registered 17 October 2013.

## Background

Domestic violence and harmful alcohol consumption are two key public health problems worldwide [1]. In addition to their high prevalence, both phenomena result in considerable biological, psychological, and sociological repercussions for individuals and the general population [1, 2]. These phenomena often co-occur and share a complex set of psychosocial risk factors. However, in-depth studies aimed at supporting interventions to address both phenomena in an integrated manner remain scarce, especially among specific groups such as Latin American women.

### Alcohol consumption among women

Although alcoholic beverage consumption remains higher among men, women have significantly increased their consumption because of society’s changing gender roles [3]. According to the Global Burden of Diseases, Injuries, and Risk Factors Study in 2010 [4], alcohol consumption is the third leading risk factor for illnesses and disabilities worldwide; furthermore, the World Health Organization (WHO) considered it to be the primary risk factor for “disability-adjusted life years” (DALYs) in Tropical Latin America, which encompasses certain Latin America countries including Brazil. Depending on consumption patterns, alcohol can affect individual’s health due to accidents, dependence, liver cirrhosis, cancer, and injury. Alcohol consumption can also affect the health of other people in community and family settings, specifically due to drinking and driving situations, work absenteeism, drinking during pregnancy, and cases of violence and neglect in the parent–child relationship [2].

Previous Brazilian surveys suggest that approximately 75 % of the population has consumed alcohol at least once in their lifetime [5], and nearly 25 % of respondents reported at least one type of drinking-related problem. Furthermore, 3 % meet the criteria for alcohol abuse, and 9 % are alcohol dependent [6]. These rates varied based on gender, age, marital status, educational level, income, and country region with approximately 4 % of women abusing alcohol or being alcohol dependent [7].

It has been suggested [3] that biological factors such as lower body weight and lower fat-to-muscle ratios contribute to faster and more intense effects of alcohol among women. Compared with men, harmful alcohol consumption among women has a disproportionate effect on their lives and health, including undesired consequences regarding reproductive function and pregnancy as well as the faster development of physical, cognitive, social, and psychiatric problems [3, 8, 9].

### Domestic violence

Like alcohol consumption, violence (specifically, domestic violence) has been identified as a priority for WHO actions. Violence is a major public health problem worldwide because it has serious implications for health (in both the short and long term) as well as the psychological and social development of individuals, families, and communities [10, 11].

Domestic violence (or family violence) can be understood as “every act or omission committed by some family member in a position of power, regardless of where it occurs, which harms the well-being, physical or psychological integrity, or freedom and right to full development of another family member” [12]. Violent acts can be classified based on their type, including acts of physical, psychological, and sexual violence; alternatively, they can be expressed as forms of neglect or abandonment [11]. In the family setting, violence can occur within interpersonal relationships, including those with children, adolescents, men, women, and the elderly [13–15]. Despite the increased emphasis placed on victimized women within partnerships [11], researchers recognize that women can also be the aggressor in the marital relationship [13, 16, 17] and with regard to their children [18, 19].

Some studies suggest that domestic violence is highly prevalent and has a complex network of associations. Some of the factors associated with domestic violence include younger age, lower education level, a history of physical violence in the family, childhood sexual abuse, depression, poor socioeconomic conditions, and problems related to alcohol consumption among one or both members of the couple [20–23]. The consequences of domestic violence can be identified at different levels of life, leading to physical and psychosocial impairments. [24, 25]. Therefore, it becomes important to treat family violence as a complex phenomenon, one that tends not to be restricted to a single member of the family [22].

### Domestic violence and alcohol

Although many studies relate alcohol consumption to violent behavior, it is not possible to establish a simple and unidirectional association because of the complexity of this relationship [1, 26–29]. A population-based study in Brazil found that the aggressor was under the influence of alcohol at the time of the event in over half of the identified cases of domestic violence [14]. The literature includes several other studies that suggest an association between alcohol consumption and violent behavior among intimate partners [1, 30–35].

Although some studies have suggested that alcohol consumption is more associated with the severity of violence acts than to increases in its occurrence [31, 36], others have suggested that it should be related to both [35] and that the association between alcohol and violence becomes more powerful with increased consumption [26, 29]. To better understand how alcohol consumption might be related to a greater severity of domestic violence, it is important to consider not only the pharmacological effects of alcohol but also the environmental and sociocultural factors that influence patterns of alcohol consumption and violent behaviors [31].

Alcohol is a risk factor for the occurrence of violent acts because it has direct effects on physical and cognitive performances, contributing to violence by reducing self-control as well as reducing judgment and the ability to recognize signs of danger. Moreover, individual and cultural beliefs that alcohol causes aggression can lead to its intake in preparation or as an excuse/justification for violent acts [1]. It is also possible that harmful alcohol consumption is a coping strategy adopted by victims to address the stress caused by violent situations [1, 37].

Although strong associations seem exist between domestic violence and alcohol abuse among women, few studies have been conducted regarding the peculiarities of this association, especially in developing countries such as Brazil. Given this association, the WHO recommends measures to reduce the availability and the harmful consumption of alcohol as important strategies to prevent violence. There is some evidence on the success of brief interventions (BIs) in reducing various forms of violence, including domestic violence [1].

### Early detection and intervention for harmful alcohol consumption

The early detection of alcohol abuse is essential in preventing the future consequences of this consumption, because it enables the early application of interventions [38–40]. Given that many users do not recognize they have a problem in the early stages, interventions that allow problems to be recognized and behaviors to be changed become necessary.

BIs that are targeted on primary or secondary prevention strategies among alcohol consumers focus on changing an individual’s behaviors through limited-time therapies that can be performed by professionals with different backgrounds [39, 41]. Despite the variations in the forms of BIs application (e.g., in person, by phone, or online), the theoretical framework of motivational interviewing [42] and the principles of cognitive-behavioral therapy are usually employed.

Because BIs are an effective, low-cost strategy for treating problems related to alcohol and drugs, it is also a useful tool for prevention and a way to facilitate the referral of severe cases (e.g., those with alcohol dependence) to specialized treatments [38, 41, 43]. Previous studies have suggested that BIs work for different types of patients and that their effectiveness can be equal to or even better than those of interventions that require more time to complete [44]. Although BIs have been shown to be effective when performed by skilled professionals or researchers [41, 45], in clinical settings or in the context of routine health services, there is a lack of studies regarding the use of these techniques in community household settings. Such an approach should reach populations who do not have access to or choose not seek out the healthcare system; furthermore, it represents an alternative to the difficulties of implementing BIs in the healthcare services.

A comprehensive review of the BIs applied in primary care concluded that this approach decreases alcohol use among men; however, these findings were not extended to women. Therefore, additional research regarding the most effective components of such interventions is needed among this specific population [43]. Moreover, another literature review [46] identified promising results for women, despite the significant heterogeneity observed among the analyzed clinical trials. Because the evidence for the effectiveness of BIs remains contradictory among women [43, 46, 47], a well-conducted study that establishes the effectiveness of BIs is necessary for this population group, especially within developing countries. The assessment of BIs in community household settings is especially relevant in Brazil because this country has a strong strategy for primary health services that is focused on providing home care to families.

The current protocol is designed to study the association between alcohol consumption and patterns of domestic violence among women. Study 1 aims to (1) estimate the rates of harmful alcohol consumption and domestic violence (within the couple and that directed toward children) among adult women; (2) assess the association between the sociodemographic and mental health factors with regard to the harmful consumption of alcoholic beverages; and (3) assess the association between the sociodemographic and physical and mental health factors as well as between alcohol consumption and the occurrence of domestic violence (within the couple and that directed toward children).

Nested within Study 1, Study 2 aims to evaluate the effectiveness of BIs on women with harmful alcohol consumption living in a community household setting in reducing their alcohol consumption and patterns of domestic violence (within couples and toward children).

## Methods

### Study design

Study 1 will employ a cross-sectional observational design to investigate the patterns of alcohol consumption and domestic violence using a household survey of women. Study 2 will be a randomized clinical trial (RCT) nested within Study 1 (i.e., the women identified as having harmful alcohol consumption in Study 1 will be invited to take part in the RCT.

### Setting

The current study will be conducted in Juiz de Fora, Minas Gerais, southeastern Brazil; this city has approximately half a million inhabitants. The study will randomly select women living in two neighborhoods with different average monthly incomes per capita according to the Instituto Brasileiro de Geografia e Estatística (Brazilian Institute of Geography and Statistics; IBGE) census conducted in 2000. This categorization will be used to investigate two populations with different income profiles.

### Sampling

To calculate the sample size needed for this cross-sectional study, the data from a previous study (which estimated the prevalence of harmful alcohol consumption among women in Juiz de Fora as approximately 9.1 %) will be considered [48]. In addition, this study considered a maximum measurement error of 3 %, 95 % confidence intervals, a finite population correction based on the number of women residing in the randomly selected neighborhoods (consistent with the 2000 IBGE census), an effect of the sampling design equal to 2, and a 25 % refusal to participate rate. Thus, the initial sample size was estimated as 824 respondents (412 in each neighborhood).

To calculate the clinical trial sample size, the following considerations will be made: (i) a significance level of 5 %; (ii) a statistical power of 80 % (to compare proportions of the two independent groups); (iii) a non-interview rate of 15 % due to refusal to participate or failure to locate the informant; (iv) the effect of the BI after 3 months (i.e., an expected reduction of at least 20 % in the number of women with harmful alcohol consumption; and (v) an effect of the sampling design equal to 2. Thus, the calculated sample size for each group (i.e., the treated group [TG] and control group [CG]) will be 73 women.

To make the second study possible, considering the prevalence of women with risky alcohol consumption in the two neighborhoods surveyed around the estimated above (ie, 9.1 %), there will be necessary an increase in the sample size for Study 1, which now has approximately 1600, with 800 in each of the neighborhoods.

To recruit the women who belong to the target population, a two-stage cluster sampling method will be adopted. The streets belonging to each neighborhood will be considered as primary sampling units (PSUs), and households will be considered as secondary sampling units (SSUs). To select PSUs, a simple random sampling method without replacement will be adopted, whereas the SSUs will be selected using systematic sampling. In each selected household, the field researchers will invites all women who met the inclusion criteria to participate in the study.

### Inclusion criteria

Female residents in the two neighborhoods who are aged 18–60 years, literate, and without obvious cognitive impairments will comprise the target sample. Domestic violence behaviors will be investigated among women who have children of aged up to 18, husbands or partners, or both residing in the same household.

### Measurements

#### Participant sociodemographic characteristics

Information concerning ethnicity, age, education level, religion, occupation, marital status, and number of children will be obtained. Among women who are married or cohabitating, information regarding the duration of their relationship will be obtained along with the sociodemographic characteristics of their partners (e.g., age, education level, and occupation).

#### Alcohol consumption

Alcohol consumption will be assessed using the Alcohol Use Disorders Identification Test (AUDIT), a screening tool developed by the WHO [49] that consists of 10 questions: 3 concerning consumption amount and frequency, 3 regarding dependence symptoms, and 4 concerning personal and social problems related to alcohol abuse. The final score reflects the following alcohol consumption levels or patterns: abstainers/low risk (0–7 points), hazardous use (8–15 points), harmful use (16–19 points), and probable dependence (20–40 points). The AUDIT has been previously validated in several countries, including Brazil, and shows good sensitivity (mean = 0.90) and specificity (mean = 0.80) regarding the detection of harmful alcohol consumption [49, 50].

Information concerning the respondents’ use of any healthcare service over the previous 3 months, the approach of health professionals regarding respondents’ alcohol consumption, respondent participation in any treatment for alcohol consumption, and the respondents’ perceptions of their partners’ alcohol consumption will also be obtained.

#### Consumption of other substances

Information regarding the consumption of tobacco, marijuana, amphetamines, benzodiazepines, antidepressants, and cocaine over the three months preceding the interview will be obtained.

#### Health

A structured 15-question instrument concerning the respondents’ general health and the presence of specific diseases such as hypertension, diabetes, and heart problems will be administered.

#### Depression

The population-based screening scale for depression from the Center for Epidemiologic Studies (i.e., the CES-D) will be used. This screening instrument seeks to identify depressive symptoms within adult population-based studies [51]. Consisting of 20 items, its total score ranges from 0 to 60 points, with the higher scores indicating a greater amount of depressive symptoms. The cutoff of 16 points is often used to classify individuals as having possible depression. The CES-D has been previously validated for use in Brazil and shows satisfactory levels of reliability [52, 53].

#### Domestic violence

The Revised Conflict Tactics Scales (CTS2) will be employed to collect information on violence between intimate partners, whereas the Parent–child Conflict Tactics Scales (CTSPC) will be used to assess domestic violence by women against their children.

The CTS2 was designed to assess violence within couples, and it provides data on the respondent and her partner. This survey consists of 78 items, and each item is displayed in a set of two questions. The first question of each set refers to a possible behavior of the respondent, whereas the second refers to the same action but experienced by the partner. This instrument consists of 5 subscales that address the occurrence of negotiation, psychological aggression, physical violence, consequences of violence affecting the health of the respondent and her partner (i.e., injuries), and sexual coercion within the relationship. The CTS2 shows an internal consistency between 0.65–0.86 and an intra-observer reliability (kappa) above 0.75 when evaluated in Brazilian samples to assess its conceptual equivalence, semantic equivalence, and psychometric properties [54–56].

The CTSPC was designed based on the refinement of the previous instrument, and it addresses the parent–child relationship. This instrument is composed of questions regarding the occurrence of certain behaviors directed at children. Its 22 items are divided into three levels: nonviolent discipline (e.g., explaining errors and applying punishment), psychological aggression (e.g., swearing, screaming, threatening to kick the child out, or hitting), and physical assault (e.g., face slapping, throwing the child on the floor, or threatening with a knife); the latter was subdivided into corporal punishment, physical abuse, and severe physical abuse. The CTSPC had previously been cross-culturally adapted for use in Brazil; furthermore, prior work found that its internal consistency ranged from 0.49–0.68, and estimates of its intra-observer reliability (kappa) were above 0.75 [57, 58].

According to Straus [59] (the author of the CTS2 and CTSPC), these instruments investigate the occurrence of behaviors that, unlike emotions, attitudes, and beliefs, are less susceptible to distortions with regard to the interpretation of facts. Importantly, the methodological choice for these assessments reflects an understanding of domestic violence as a complex phenomenon that tends not to remain restricted to only one member of the family [58]. Furthermore, research indicates that violent acts are typically reciprocal, i.e., the respondent might be both the aggressor and the victim [60].

The information regarding alcohol consumption provided by the respondent and her partner during episodes of violence will also be incorporated by adapting the violence scales. After each CTS2 and CTSPC item, the interviewer will ask whether one of the involved individuals was under the influence of alcohol when the event occurred.

The CTS2 and CTSPC will be self-applied, whereas all of the other instruments will be administered to participants during interviews. Except for the CES-D, which refers to the events of the previous week, all of the instruments will be adapted to consider a time period of three months.

### Procedure

#### Study 1: Household survey

A researcher will approach the participants at their homes; all of the instruments will be administered there. The interviews will take approximately 30 min to complete and will be held in a place as private as possible to allow the interviewees to freely answer questions and increase the credibility of their answers.

The researchers will be properly trained women who will initially approach the participants to invite them to volunteer for the survey, delivering a document that describes the study and its objectives. The training of the team will consist of attending lectures on domestic violence, alcohol consumption, BIs, the project details, a clarification of the study’s procedures, and an explanation of the questionnaires. Furthermore, the importance of research ethics will be emphasized. The researchers will gain familiarity with the instruments by administering them to a colleague. Training will be provided on the application of a BI via videos and role-playing techniques.

To locate the women living in the randomly selected households, at least three visits will be made to each household. When a resident is not found at her supposed address, the researchers will seek information from the neighbors to identify the existence of any women who meet the eligibility criteria for the study and the best days and times to approach them.

#### Study 2: Random clinical trial

The data collection for Study 2 will be conducted concomitantly using the women detected in Study 1. Women with AUDIT scores equal to or above 8 will be considered as hazardous alcohol users and randomly allocated into one of two groups: the TG or the CG. The TG will receive a single BI after the instrument is administered, whereas the CG will not receive BI until after the end of the study.

The participants will be allocated using a list of random numbers. Three months after the BI, all instruments except for the sociodemographic questionnaire will be re-administered to both groups. Because of ethical reasons, a BI session will also be offered to the CG after the second evaluation, at the end of the study. Independent interviewers will perform the baseline and the follow-up interview.

#### The intervention

The BI will be performed at the participants’ households in accordance with the model proposed by Miller and Sanchez [61], the principles of which are summarized by the acronym “FRAMES”. This acronym stands for “Feedback” (provide information regarding the score obtained on the screening instrument), “Responsibility” (the patient must take responsibility for changing their behavior), “Advice” (guidance on issues related to the use of substances and coping strategies), “Menu of options” (a list of options or alternative behaviors to substance use), “Empathy” (putting one’s self in another’s shoes based on one’s own assumptions to try to understand their behavior), and “Self-efficacy” (patients’ belief in their ability to change).

The timing of the BI will be controlled, ranging from 15 to 40 min. After administering the procedure, the researcher will record the approach used and the participant’s behavior during the intervention.

### Ethics

This study has been approved by Ethics Committee of the Federal University of São Paulo (Universidade Federal de São Paulo; UNIFESP- registration number 0699/10). The study has also been registered in the Brazilian Clinical Trials Registry (ReBEC; registration number: RBR-7rjt4t).

All participants will receive information about the study, and their participation will be voluntary. Each participant will be required to sign an informed consent document. At the end of the interviews, all participants will receive an informational leaflet regarding the use of alcoholic beverages, as well as a guide concerning health services and psychosocial assistance in cases of violence and alcohol dependence. After the follow-up assessment is performed three months after the screening, the BI will be applied to participants in the CG. Women who score in the range of “probable alcohol dependence” (i.e., 20 points or more on the AUDIT) will be instructed to seek specialized treatment, regardless of the group to which they are allocated.

### Statistical analyses

Given the characteristics of the adopted sampling design, the data will be processed and analyzed using the statistical software STATA version 11.

Initially, exploratory bivariate analyses will be conducted to assess the associations between the variables of interest and the possible risk factors. Chi-square tests will be used to test the association between categorical variables, whereas Student’s *t*-test will be used to compare the means of continuous variables.

A logistic regression model will be used to examine the patterns of alcohol consumption with the response variable and the sociodemographic and health characteristics as explanatory variables. A separate logistic regression model will be used with the type of violence as dependent variable and the sociodemographic and health characteristics, and alcohol consumption patterns as explanatory variables.

The TG and CG will be compared using t-tests and generalized linear models with repeated-measures.

## Discussion

Both alcohol consumption among women and violence within the family are relevant public health problems which are mediated by cultural and contextual factors [20]. Despite the purported associations between these phenomena, studies providing information on their characteristics among women using community samples are scarce in Brazil [19, 21, 62].

This study includes a household survey with representative samples of two neighborhoods with different socioeconomic profiles in Brazil, a developing country where well-conducted household surveys remain rare. Although the cross-sectional design does not allow for the inference of causality, this approach will broaden the discussion on the relationship between alcohol consumption and violence, considering the individual and environmental aspects involved in these phenomena.

This study not only proposes to present a better understanding of violence in the lives of women (regardless of its association with alcohol consumption) but also expand the discussion regarding the possible public healthcare strategies and actions needed to prevent domestic violence and harmful alcohol consumption [1]. With regard to alcohol, the use of a BI in a community household setting represents a new method of prevention that could have indirect effects on domestic-violence-related problems.

We believe that this protocol has the potential to support supplementary studies aiming to promote knowledge regarding two of the most important public health problems from different perspectives: harmful alcohol consumption and domestic violence. Furthermore, we believe that the results of this study might influence both clinical practice and prevention efforts in the context of public health.

## Abbreviations

AUDIT: Alcohol use disorders identification test
BI: Brief intervention
CES-D: Center for epidemiologic studies depression scale
CTS2: Revised conflict tactics scales
CTSPC: Parent–child conflict tactics scales
DALYs: Disability-adjusted life years
IBGE: Brazilian institute of geography and statistics
WHO: World health organization
